# Association of pigmentation related-genes polymorphisms and geographic environmental variables in the Chinese population

**DOI:** 10.1186/s41065-021-00189-7

**Published:** 2021-07-08

**Authors:** Yuxin Wang

**Affiliations:** Queen Mary School, Nanchang University, 461 Bayi Road, Nanchang, 330006 Jiangxi China

**Keywords:** Association study, Polymorphisms, Environmental factors, Selection pressure

## Abstract

**Background:**

Human skin color is highly heritable and one of the most variable phenotypic traits. However, the genetic causes and environmental selective pressures underlying this phenotypic variation have remained largely unknown. To investigate whether the pigmentation related-genes polymorphisms are associated with the geographic environmental variables. We selected randomly 795 healthy individuals from eight ethnic groups in nine provinces in China. Six single nucleotide polymorphisms (SNPs) of *SLC45A2* and *TYR* were genotyped using Agena MassARRAY. The Chi-square test and Spearman correlation analysis were used to compare the frequency distribution of genotypes among different ethnic groups and evaluate the relationship between SNP genetic diversity and environmental variables, respectively.

**Results:**

The results indicated that rs28777 and rs183671 (*SLC45A2*) and rs1042602 (*TYR*) genotype frequency distributions were significantly different between the Xinjiang-Uighur and other ethnic groups (*P* < 0.05). Spearman correlation analysis found that rs28777-A (*r* = − 0.090, *P* = 0.011), rs183671-G (*r* = − 0.105, *P* = 0.003), rs1042602-A (*r* = − 0.108, *P* = 0.002), rs1126809-A (*r* = − 0.151, *P* < 0.001) allele frequencies were negatively correlated with the longitude; rs183671-G (*r* = 0.151), rs1042602-A (*r* = 0.157) and rs1126809-A (*r* = 0.138) allele frequencies were positively associated with the latitude (*P* < 0.001); rs183671-G (*r* = 0.116, *P* = 0.001), rs1042602-A (*r* = 0.105, *P* = 0.003) and rs1126809-A (*r* = 0.070, *P* = 0.048) allele frequencies were positively correlated with the sunshine hours; rs183671-G (*r* = − 0.076, *P* = 0.033), rs1042602-A (*r* = − 0.079, *P* = 0.027) and rs1126809-A (*r* = − 0.076, *P* = 0.031) were negatively correlated with the annual average temperature.

**Conclusions:**

Our results confirmed the idea that environmental factors have been an important selective pressure upon pigmentation related gene polymorphisms.

**Supplementary Information:**

The online version contains supplementary material available at 10.1186/s41065-021-00189-7.

## Introduction

Human skin color is highly heritable and one of the most variable phenotypic traits that can vary dramatically within and across ethnic populations [1]. It is known that the human skin color is predominantly determined by pigments include melanin, hemoglobin (red), hemosiderin (brown), carotene (yellow), and bilirubin (yellow) [2]. Among those, the amount, type, and distribution of melanin play key roles in determining human skin pigmentation. Studies indicate that the human skin pigmentation in global populations is highly associated with latitude, and fundamentally, the distribution of ultraviolet (UV) radiation [3, 4]. Moreover, the researchers believe that geographic variation in skin pigmentation was influenced by the concerted action of different types of natural selection, including climate, lifestyle, diet, metabolism [1]. However, the genetic causes and environmental selective pressures underlying this range of skin color variation have remained largely unknown.

With the rapid development of genetics and genomics, researchers have gradually realized that the human skin color diversity is due to the natural positive selection of those genes that impact on human pigmentation, especially in the melanosome biogenesis or the melanin biosynthetic pathways [5, 6]. Recently, a large number of genome-wide association studies (GWAS) for pigmentation have been established and identified that some single nucleotide polymorphisms (SNPs) on *TYR*, *IRF4*, *TYRP1*, *OCA2*, *SLC45A2*, *MC1R* and *KITLG* genes are significantly associated with human skin color [7–10]. The solute carrier family 45, member 2 (*SLC45A2*) gene encodes the membrane associated transporter protein (MATP). The SLC45A2 protein expresses in melanocyte cell lines and mediates melanin synthesis by tyrosinase trafficking and proton transportation to melanosomes [11]. *SLC45A2* mutations cause oculocutaneous albinism type IV (OCA4) and polymorphisms of *SLC45A2* gene are associated with dark skin, hair, and eye pigmentation [12, 13]. In addition, the *TYR* gene encodes tyrosinase, a multifunctional enzyme that plays a major role in melanin biosynthesis in melanocytes [14]. *TYR* is commonly known as the albino locus since the homozygous or compound heterozygous mutations of this gene result in oculocutaneous albinism type 1 (OCA1), an autosomal recessive genetic disorder characterized by hypopigmented hair, skin and eyes [15].

However, the genetic causes and environmental selective pressures underlying this range of phenotypic variation have remained largely unknown. Therefore, to investigate whether the six polymorphisms in the two pigmentation related-genes *SLC45A2* (rs11568737, rs28777 and rs183671) and *TYR* gene (rs1042602, rs1393350 and rs1126809) are associated with the geographic environmental variables, we selected randomly a total of 795 healthy individuals from eight ethnic groups in nine provinces in China, while collected the geographic environmental variables (altitude, longitude, latitude, air pressure, sunshine hours, and annual average temperature). The results of this study will improve our understanding of the impact of environmental variables in genetic differentiation and maintenance of genetic variation.

## Results

**Fig. 1:**
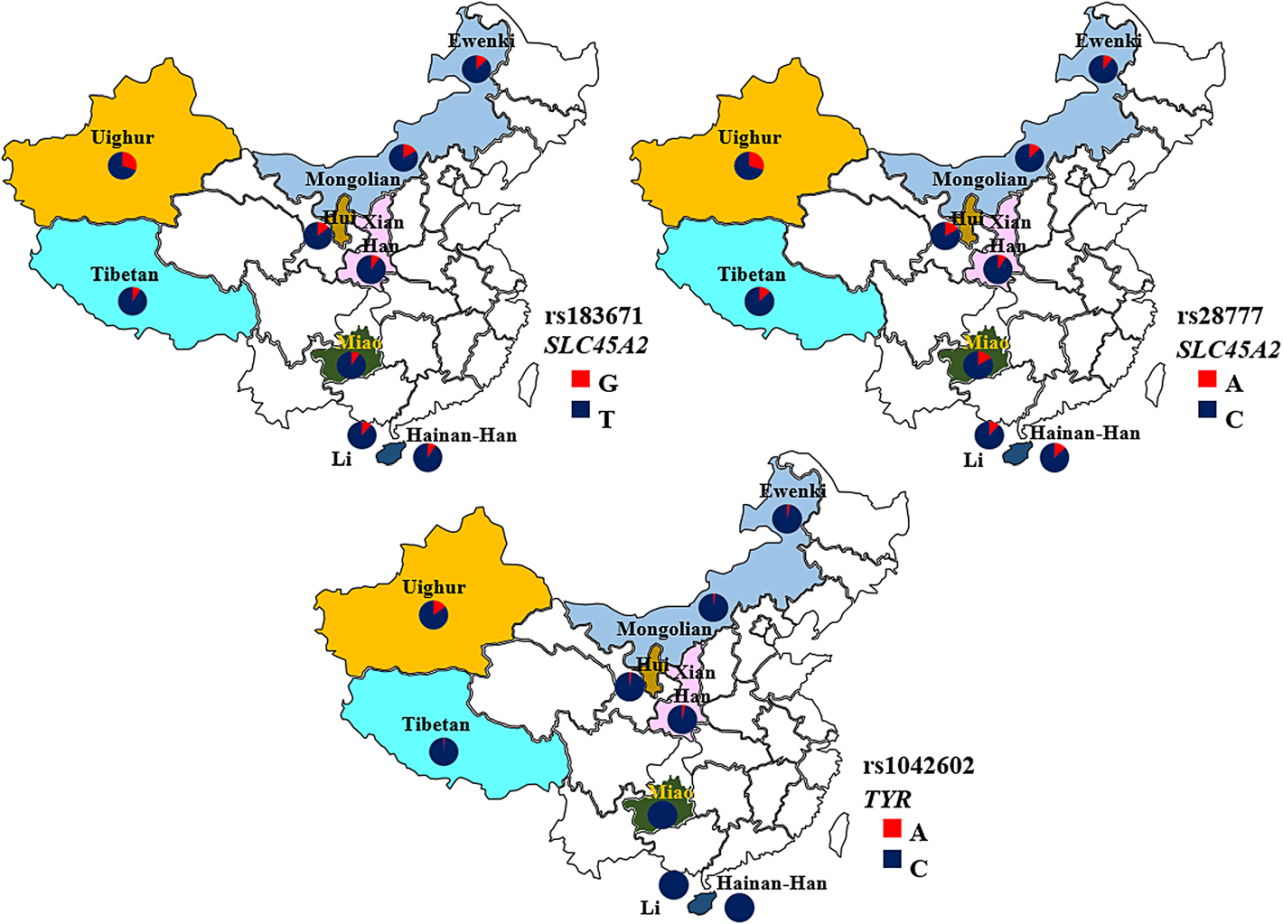
Distribution of allele frequencies of rs1042602 (A/C), rs28777 (A/C) and rs183671 (G/T) among different ethnic groups in China

A total of 795 samples including eight ethnic groups from nine provinces in China (Tibet-Tibetan accounted for 13.2%, Inner Mongolia-Ewenki 12.6%, Hainan-Han 6.2%, Ningxia-Hui 12.6%, Hainan-Li 12.5%, Inner Mongolia-Mongolian 12.6%, Guizhou-Miao 11.2%, Xinjiang-Uighur 13.3%, and Shaanxi-Han 5.9%) were collected to study the relationship between skin pigmentation-related gene variants and environmental variables. We also collected the detailed geographical environment information of different ethnic regions (Fig. 1), including altitude (m), longitude (°), latitude (°), atmosphere pressure (kPa), sunshine duration (hours), and year-round average temperature (°C), as shown in Table 1.

**Table 1:** Detailed geographical environment information of different ethnic regions

Ethnic	Residence	N	Altitude (m)	Longitude (°)	Latitude (°)	Atmosphere pressure (kPa)	Sunshine duration (hours)	Year-round average temperature (°C)
Tibetan	Naqu	4	4505	92.058	31.482	58	2879	11
	Linzhi	9	2994	94.368	29.655	70	2005	11
	Shannan	13	3572	91.78	29.243	65	2800	10
	Shigatse	20	3844	88.887	29.273	63	3248	8
	Lhasa	59	3651	91.129	29.659	64	3055	10
	Total	105						
Ewenki	Ewenki Autonomous Banner Huisumuhakemugacha	10	690	119.172	48.379	93	2900	3
	Yiminhe Town, Ewenki Autonomous Banner	19	673	119.791	48.583	93	2900	3
	Bayantuohai Town, Ewenki Autonomous Banner	21	617	119.762	49.143	94	2900	3
	Dayan Town, Ewenki Autonomous Banner	23	682	120.558	49.237	93	2900	3
	Ewenki Autonomous Banner, Xinihe East Sumu	27	788	120.3	48.867	92	2900	3
	Total	100						
Hainan-Han	Haikou City, Hainan	49	9	110.339	20.035	101	2041	24.4
Hui	Haiyuan County, Zhongwei City, Ningxia	5	1841	105.65	36.571	81	1609	11
	Guyuan City, Ningxia	10	1778	106.249	36.022	82	1602	9
	Tongxin County, Wuzhong City, Ningxia	85	1316	105.816	36.986	86	1690	12.5
	Total	100						
Li	Wangxia Town, Changjiang, Hainan	5	357	109.157	19.009	97	2300	26
	Baoting Li and Miao Autonomous County	8	54	109.707	18.647	101	2300	26
	Changjiang Li Autonomous County, Hainan	38	140	109.062	19.304	100	2300	26
	Qicha Town, Changjiang, Hainan	48	107	109.062	19.118	100	2300	26
	Total	99						
Mongolian	Chenqiba Town, Inner Mongolia	19	597	119.446	49.334	94	3205	7.7
	Hohhot	81	1056	111.668	40.819	89	2588	7.3
	Total	100						
Miao	Gaopo Township, Huaxi District, Guiyang City	39	1459	106.819	26.302	85	1060	14.8
	Mengguan Township, Huaxi District, Guiyang City	50	1196	106.755	26.415	88	1060	14.8
	Total	89						
Uighur	Bazhou	23	944	86.152	41.77	90	2990	11.5
	Ili	23	646	81.331	43.923	94	2977	5.8
	Aqsu	30	1109	80.314	41.15	89	2911	12.5
	Kashgar	30	1298	75.996	39.476	87	2760	13
	Total	106						
Shaanxi-Han	Yan’an	1	1070	109.496	36.591	89	2056	15.5
	Hancheng	1	457	110.449	35.483	96	2056	15.5
	Fuping	2	520	109.364	34.95	95	2056	15.5
	Weinan	3	355	109.516	34.506	93	2056	15.5
	Xi’an	40	381	108.947	34.27	96	2056	15.5
	Total	47						

The six SNPs on the two skin pigmentation-related gene *SLC45A2* (rs11568737, rs28777 and rs183671) and *TYR* (rs1042602, rs1393350 and rs1126809) were successfully genotyped from 795 samples (call rate > 95%). The basic information (SNP-ID, chromosome number, position, alleles and gene name) and polymerase chain reaction (PCR) primer sequence (1st-PCRP, 2nd-PCRP and unique base extension primer sequence (UEP-SEQ) of the six SNPs was showed in Table 2. The minor allele frequency (MAF), genotype frequency and Hardy-Weinberg equilibrium (HWE)-*P* value of each SNPs are shown in Supplementary Table 1, Tables 2, and 3, respectively. The results showed that except for rs1393350 in *TYR* was not in accordance with the HWE in Uighur (*P* < 0.01), other five SNPs were in accordance with the HWE in the nine groups (*P* > 0.01).

**Table 2:** The basic information and primer sequence of SNPs

SNP-ID	Chromosome	Position	Alleles	Genes	1st-PCRP	2nd-PCRP	UEP_SEQ
rs11568737	5	33,944,743	T > C	*SLC45A2*	ACGTTGGATGGTGATCACCACGACGACAAC	ACGTTGGATGATGGTGCAGCTGGCTCAGAT	gGGGCTTTCTGGTCAAC
rs28777	5	33,958,854	C > A	*SLC45A2*	ACGTTGGATGAAAAGGCTTCCACTCAGTTG	ACGTTGGATGCAAGAGTCGCATAGGACAGG	cctcCGTCCCATCCACTCAGAG
rs183671	5	33,964,105	T > G	*SLC45A2*	ACGTTGGATGTCCTCATGCATAGACACTCC	ACGTTGGATGATATCCAGGTTGCCTCTGCT	ggcaTCTGCTGTCTTCAGGG
rs1042602	11	89,178,528	C > A	*TYR*	ACGTTGGATGTGACCTCTTTGTCTGGATGC	ACGTTGGATGGGTGCTTCATGGGCAAAATC	TCAATGTCTCTCCAGATTTCA
rs1393350	11	89,277,878	G > A	*TYR*	ACGTTGGATGGCATATCCACCAACTCCTAC	ACGTTGGATGGGAAGGTGAATGATAACACG	TTTGTAAAAGACCACACAGATTT
rs1126809	11	89,284,793	G > A	*TYR*	ACGTTGGATGAATGGGTGCATTGGCTTCTG	ACGTTGGATGCCTCTGCAGTATTTTTGAGC	catcTTGAGCAGTGGCTCC

*SNP* single nucleotide polymorphism, *Chr* chromosome, *PCRP* polymerase chain reaction primer, *UEP_SEQ* unique base extension primer sequence

**Table 3:** Differences in genotype distributions of SNPs among different ethnic groups

SNP-ID	Ethnic	Ewenki	Hainan-Han	Hui	Li	Miao	Mongolian	Tibetan	Uighur	Shaanxi-Han
rs28777	Ewenki									
	Hainan-Han	0.811								
	Hui	0.279	0.561							
	Li	0.977	0.897	0.323						
	Miao	0.248	0.763	0.271	0.346					
	Mongolian	0.778	0.998	0.353	0.887	0.614				
	Tibetan	0.783	0.995	0.318	0.891	0.591	0.999			
	Uighur	1.12E-05	5.32E-03	9.27E-04	2.87E-05	6.78E-03	1.65E-04	1.09E-04		
	Shaanxi-Han	0.617	0.557	0.209	0.617	0.278	0.560	0.576	5.02E-04	
rs183671	Ewenki									
	Hainan-Han	0.416								
	Hui	0.366	0.545							
	Li	0.608	0.837	0.662						
	Miao	0.503	0.322	0.155	0.401					
	Mongolian	0.506	0.142	0.265	0.197	0.102				
	Tibetan	0.575	0.784	0.301	0.797	0.620	0.098			
	Uighur	3.20E-05	5.18E-05	4.80E-05	3.29E-06	1.43E-06	2.46E-03	2.59E-07		
	Shaanxi-Han	0.675	0.516	0.370	0.616	NA	0.252	0.769	2.04E-04	
rs1042602	Uighur	3.53E-04	3.71E-04	4.56E-05	3.05E-07	1.09E-06	4.56E-05	2.19E-06		1.58E-02

*SNP* single nucleotide polymorphism

*P* < 0.05 was considered to be significant

In addition, we used the Chi-square test to evaluate the difference of genotype frequency distribution of the five SNPs among eight ethnic groups, as shown in Table 3. The results indicated that the genotype frequency distribution of rs28777 and rs183671 (*SLC45A2*) and rs1042602 (*TYR*) were significantly different between the Xinjiang-Uighur and other ethnic groups (*P* < 0.05). The allele frequency distribution of these three significantly different SNPs was shown in Fig. 1.

Simultaneously, we analyzed the relationship between SNP genetic diversity and environmental variables using Spearman correlation analysis (Table 4). It was found that the allele frequencies of rs28777-A (*r* = − 0.090, *P* = 0.011), rs183671-G (*r* = − 0.105, *P* = 0.003), rs1042602-A (*r* = − 0.108, *P* = 0.002), rs1126809-A (*r* = − 0.151, *P* < 0.001) were negatively correlated with the longitude. However, the positive correlation between the alleles frequencies of rs183671-G (*r* = 0.151), rs1042602-A (*r* = 0.157) and rs1126809-A (*r* = 0.138) and the latitude were extremely significant (*P* < 0.001). The alleles frequencies of rs183671-G (*r* = 0.116, *P* = 0.001), rs1042602-A (*r* = 0.105, *P* = 0.003) and rs1126809-A (*r* = 0.070, *P* = 0.048) were found to be significantly positively correlated with the sunshine hours. However, the alleles frequencies of rs183671-G (*r* = − 0.076, *P* = 0.033), rs1042602-A (*r* = − 0.079, *P* = 0.027) and rs1126809-A (*r* = − 0.076, *P* = 0.031) were significantly negatively correlated with the annual average temperature. The correlations between the allele frequencies of other SNPs and environmental variables were not significant. These findings indicate that environmental factors have selective pressure on these SNPs.

**Table 4:** The association between polymorphisms and geographic environmental variables

SNP-ID	Altitude		Longitude		Latitude		Air pressure		Sunshine hours		Annual average temperature	
	γ	p	γ	p	γ	p	γ	p	γ	p	γ	p
rs11568737	0.038	0.286	−0.031	0.383	−0.007	0.851	−0.037	0.302	0.010	0.783	0.011	0.748
rs28777	0.002	0.958	−0.090	0.011	0.036	0.313	0.003	0.942	0.036	0.305	−0.011	0.761
rs183671	−0.002	0.950	−0.105	0.003	0.151	1.86E-05	0.006	0.872	0.116	0.001	−0.076	0.033
rs1042602	0.004	0.903	−0.108	0.002	0.157	8.54E-06	0.005	0.893	0.105	0.003	−0.079	0.027
rs1126809	0.022	0.539	−0.151	1.90E-05	0.138	9.38E-05	−0.015	0.666	0.070	0.048	−0.076	0.031

*P* < 0.05 was considered to be significant

## Discussion

To investigate whether the two pigmentation related genes (*SLC45A2* and *TYR*) polymorphisms are associated with the geographic environmental variables (altitude, longitude, latitude, and air pressure, sunshine hours, and annual average temperature), we selected randomly selected 795 healthy individuals from eight ethnic groups in nine provinces in China. The results of this study found that the genotype frequency distribution of rs28777 and rs183671 in *SLC45A2* and rs1042602 in *TYR* were significantly different between the Xinjiang-Uighur and other ethnic groups (*P* < 0.05). Simultaneously, the rs28777, rs183671, rs1042602, rs1126809 polymorphisms were found to be correlated with the geographic environmental variables (longitude, latitude, sunshine hours or annual average temperature).

*SLC45A2* (as also AIM1 or MATP) encodes a transporter protein that mediates melanin synthesis, which is expressed in a high percentage of melanoma cell lines. It has been reported that some *SLC45A2* mutations cause OCA4 and polymorphisms of this gene were found to be significantly associated with human skin, hair, and eye pigmentation, and its mutation frequency varies significantly among the global population. Yuko Abe et al. found that rs11568737 in *SLC45A2* (T500P) was significantly associated with melanin index [16]. A multi-stage GWAS of natural hair color in European ancestry found that rs28777 (*SLC45A2*) was associated with skin color and tanning ability [17]. A large Australian population-based case control study reveal that rs28777 exhibited the strongest crude association with risk of cutaneous malignant melanoma [18]. The study found that rs183671 (*SLC45A2*) was in strong linkage disequilibrium (LD) with rs16891982 (F374L) in CEU. A previous GWAS declared that the frequency of the rs183671 derived allele increased from Southern to Northern Europe, and this SNP was associated with skin pigmentation, and that each copy of the derived allele lightens the skin by 1.2 M index units [19]. Moreover, a previous GWAS demonstrated that the SNP rs183671 can explain skin color variation in three European studies RS, BTNS, and TwinsUK [20].

*TYR* is located at human chromosome 11q14.3, and encodes tyrosinase, which regulates the biosynthesis of melanin. Previous study demonstrated that mutations in *TYR* can cause OCA1 [15]. The non-synonymous polymorphism rs1042602 (Ser192Tyr) in *TYR* derived allele has specifically high frequency in Europe, and rs1042602 was significantly associated with eye color, freckles and lighter skin pigmentation [21–24]. It has been reported that rs1393350 was also associated with human hair, eye and skin color and tanning ability [23, 25–27]. A GWAS of melanoma conducted by the GenoMEL consortium identifies the locus rs1393350 associated with melanoma risk [28]. The rs1126809 variant is located in exon 4 of *TYR* gene and encodes a tyrosinase enzyme with an arginine-to-glutamine substitution at codon 402 (R402Q), and is a strong linkage with rs1393350 [29, 30]. The mutation of rs1126809 (A-G) causes the TYR enzyme to be thermosensitive, thus less active [31]. The rs1126809 has previously been used as a marker for skin pigmentation and also influence brown eye color formation [23, 30]. Previous GWAS indicated that the allele A of rs1042602 (*TYR*) was highly associated with lighter skin color in a South Asian descent population [32]. It has reported that the allele A of rs1042602 was over-represented in the Indo­Europeans population [33]. The two polymorphisms (rs1042602 and rs1126809) in *TYR* appear at high frequency in Europeans and are largely absent in African populations [34].

This study indicated that the genotype distribution of rs28777 and rs18367 in Xinjiang-Uighur was significantly different from other ethnic groups. Moreover, the allele frequencies of rs28777-A, rs183671-G, rs1042602-A, rs1126809-A were negatively correlated with the longitude; rs183671, rs1042602 and rs1126809 allele frequencies were positively associated with the latitude and the sunshine hours, while were negatively correlated with the annual average temperature in Chinese population. At present, there are few research reports on the association between genetic polymorphism and environmental factors. In 2010, Ji et al. [35] found that the disease-predisposition polymorphisms of the melatonin receptors were associated with sunshine duration in the global human populations. These results indicated that environmental factors had selective pressure on these loci, and their changes were related to environmental variables, that is, differences in selection caused by differences in environmental factors play an important role in genetic differentiation.

However, this study has some limitations that cannot be ignored. First, the sample size is small and the statistical power is relatively low. Second, this study is the first to explore the correlation between the allele frequencies of these six SNPs and geographical environmental factors. Third, we only selected six SNP loci on two genes to explore their correlation with geographical environmental factors. Finally, the effect of these genetic variations on human skin color diversity is not involved in this study. Therefore, we will further collect a larger sample and choose more SNPs and design functional experiments to explore the impact of environmental factors on genetic mutations.

In summary, this study results indicate that rs28777, rs183671 (*SLC45A2*) and 1,042,602 (*TYR*) polymorphisms were different among different populations. More importantly, our results confirm the idea that environmental factors have been an important selective pressure upon pigmentation related gene polymorphisms (rs28777, rs183671, rs1042602 and rs1126809). Further association and functional studies need to confirm our results in a large sample and explore the influence of geographical environment factors on the skin pigmentation-related genes polymorphisms and the mechanism of action.

## Materials and methods

### Study design

This study randomly selected a total of 795 healthy individuals from eight ethnic groups in nine provinces in China, including 105 Tibetan individuals, 100 Ewenki individuals, 49 Hainan Han individuals, 100 Hui individuals, 99 Li individuals, 100 Mongolian individuals, 89 Miao individuals, 106 Uighur individuals, and 47 Shaanxi-Han individuals. The basic situation of each population was shown in Table 1. The climate data (sunshine hours and annual average temperature) are quoted from China’s surface climate data in 2019. The information of altitude, longitude, latitude, and air pressure was collected through online query. Individuals who have a history of skin pigmentation-related diseases (albinism or melanoma), history of serious illness, mental illness, pregnancy were excluded from the study.

### DNA extraction

The peripheral venous blood sample (5 mL) from each subjects were taken from fasting in the morning using the Ethylene diamine tetraacetic acid (EDTA) tube, and stored at − 20 °C refrigerator for further experiment. The GoldMag-Mini Whole Blood Genomic DNA Purification Kit (GoldMag. Co. Ltd., Xi’an, China) was used to extract genomic DNA, including blood lysis, adding GoldMag® gold magnetic particles to bind DNA, magnetic separation, washing, elution, magnetic separation to obtain DNA. In order to determine the concentration and purity of the extracted DNA, we use a spectrophotometer (Nanodrop 2000, Thermo Fisher Scientific, Waltham, MA, USA). If the ratio of OD260/OD280 ratios is about 1.8, the extracted DNA is qualified.

### SNP selection and genotyping

We randomly selected the six SNPs (rs11568737, rs28777 and rs183671 in the *SLC45A2* gene and rs1042602, rs1393350 and rs1126809 in the *TYR* gene) based on previously published genes related to pigmentation [18, 21, 28, 36–40]. The online software Agena Bioscience Assay Design Suite Version 2.0 (https://agenacx.com/online-tools/) was used to design the primers sequence (Table 2). The Agena MassARRAY platform (Agena Bioscience, San Diego, CA, USA) was used to genotype the six SNPs from 795 samples, according to the manufacturer’s instructions, including DNA sample preparation; polymerase chain reaction (PCR) amplification (95 °C pre-denaturation 2 min; 45 cycles (95 °C denaturation 30s, 56 °C annealing 30s, 72 °C extension 60s); 72 °C extension 5 min; 4 °C storage); shrimp alkaline phosphatase purification; Unique base extension primer (UEP) reaction; resin purification; spotting and mass spectrometry. Genotyping results data management and analysis using the Agena Bioscience TYPER software (version 4.0).

### Statistical analysis

We used the Microsoft Excel (Microsoft Corp., Redmond, WA, USA) and Statistical Package for the Social Sciences (SPSS) version 25 (SPSS, Chicago, IL) to perform statistical analysis. The Chi-square test was used to evaluate whether each SNP was consistent with Hardy-Weinberg Equilibrium (HWE), and compare whether there are significant differences in the frequency distribution of genotypes among different ethnic groups. The relationship between SNP genetic diversity and environmental variables was analyzed using Spearman correlation analysis. All statistical analyses were two sided and the *P* < 0.05 was considered as statistically significant.

## Supplementary Information

**Additional file 1: Supplementary Table 1.** The minor allele frequency of each SNP in different ethnic groups.

**Additional file 2: Supplementary Table 2.** The genotype frequency of each SNP in different ethnic groups.

**Additional file 3: Supplementary Table 3.** The HWE-*P* value of each SNP in different ethnic groups.

## Data Availability

The data that support the findings of this study are available from the corresponding author upon reasonable request.

## Abbreviations

EDTA: Ethylene diamine tetraacetic acid
GWAS: Genome-wide association studies
HWE: Hardy-Weinberg Equilibrium
LD: Linkage disequilibrium
MATP: Membrane associated transporter protein
OCA: Oculocutaneous albinism type
PCR: Polymerase chain reaction
SLC45A2: Solute carrier family 45, member 2
SNPs: Single nucleotide polymorphisms
SPSS: Statistical Package for the Social Sciences
UV: Ultraviolet
