# Supplementary material for: Association of pigmentation related-genes polymorphisms and geographic environmental variables in the Chinese population
Source: Hereditas. 2021 Jul 8;158:24. doi: 10.1186/s41065-021-00189-7 (PMC8268332; doi:10.1186/s41065-021-00189-7)
Supplement: Supplementary file 1 — Additional file 1: Supplementary Table 1. The minor allele frequency of each SNP in different ethnic groups. [file 41065_2021_189_MOESM1_ESM.docx]

Supplementary Table 1 The minor allele frequency of each SNP in different ethnic groups

| SNP-ID | Alleles A/B | Tibetan | Ewenki | Hainan-Han | Hui | Li | Mongolian | Miao | Uighur | Shaanxi-Han |
| --- | --- | --- | --- | --- | --- | --- | --- | --- | --- | --- |
| rs11568737 | C/T | 0.038 | 0.015 | 0.000 | 0.040 | 0.040 | 0.040 | 0.028 | 0.024 | 0.053 |
| rs28777 | A/C | 0.129 | 0.110 | 0.133 | 0.165 | 0.116 | 0.130 | 0.163 | 0.297 | 0.096 |
| rs183671 | G/T | 0.095 | 0.125 | 0.092 | 0.140 | 0.111 | 0.165 | 0.096 | 0.305 | 0.096 |
| rs1042602 | A/C | 0.010 | 0.030 | 0.000 | 0.020 | 0.000 | 0.020 | 0.000 | 0.151 | 0.032 |
| rs1393350 | A/G | 0.000 | 0.010 | 0.000 | 0.035 | 0.000 | 0.025 | 0.000 | 0.099 | 0.000 |
| rs1126809 | A/G | 0.000 | 0.005 | 0.000 | 0.035 | 0.005 | 0.025 | 0.000 | 0.085 | 0.000 |

SNP: single nucleotide polymorphism
