# Supplementary material for: Association of pigmentation related-genes polymorphisms and geographic environmental variables in the Chinese population
Source: Hereditas. 2021 Jul 8;158:24. doi: 10.1186/s41065-021-00189-7 (PMC8268332; doi:10.1186/s41065-021-00189-7)
Supplement: Supplementary file 2 — Additional file 2: Supplementary Table 2. The genotype frequency of each SNP in different ethnic groups. [file 41065_2021_189_MOESM2_ESM.docx]

Supplementary Table 2 The genotype frequency of each SNP in different ethnic groups

| SNP-ID | Genotypes | Tibetan | Ewenki | Hainan-Han | Hui | Li | Mongolian | Miao | Uighur | Shaanxi-Han |
| --- | --- | --- | --- | --- | --- | --- | --- | --- | --- | --- |
| rs11568737 | CC | 0 (0%) | 0 (0%) | 0 (0%) | 0 (0%) | 1 (1%) | 0 (0%) | 0 (0%) | 0 (0%) | 0 (0%) |
|  | TC | 8 (7.6%) | 3 (3%) | 0 (0%) | 8 (8%) | 6 (6.1%) | 8 (8%) | 5 (5.6%) | 5 (4.7%) | 5 (10.6%) |
|  | TT | 97 (92.4%) | 97 (97%) | 49 (100%) | 92 (92%) | 92 (92.9%) | 92 (92%) | 84 (94.4%) | 101 (95.3%) | 42 (89.4%) |
| rs28777 | AA | 2 (1.9%) | 2 (2%) | 1 (2%) | 6 (6%) | 2 (2%) | 2 (2%) | 2 (2.2%) | 8 (7.5%) | 0 (0%) |
|  | CA | 23 (21.9%) | 18 (18%) | 11 (22.4%) | 21 (21%) | 19 (19.2%) | 22 (22%) | 25 (28.1%) | 47 (44.3%) | 9 (19.1%) |
|  | CC | 80 (76.2%) | 80 (80%) | 37 (75.5%) | 73 (73%) | 78 (78.8%) | 76 (76%) | 62 (69.7%) | 51 (48.1%) | 38 (80.9%) |
| rs183671 | GG | 1 (1%) | 1 (1%) | 1 (2%) | 4 (4%) | 2 (2%) | 2 (2%) | 0 (0%) | 7 (6.6%) | 0 (0%) |
|  | GT | 18 (17.1%) | 23 (23%) | 7 (14.3%) | 20 (20%) | 18 (18.2%) | 29 (29%) | 17 (19.1%) | 50 (47.2%) | 9 (19.1%) |
|  | TT | 86 (81.9%) | 76 (76%) | 41 (83.7%) | 76 (76%) | 79 (79.8%) | 69 (69%) | 72 (80.9%) | 48 (45.3%) | 38 (80.9%) |
| rs1042602 | AA | 0 (0%) | 0 (0%) | 0 (0%) | 0 (0%) | 0 (0%) | 0 (0%) | 0 (0%) | 4 (3.8%) | 0 (0%) |
|  | CA | 2 (1.9%) | 6 (6%) | 0 (0%) | 4 (4%) | 0 (0%) | 4 (4%) | 0 (0%) | 24 (22.6%) | 3 (6.4%) |
|  | CC | 103 (98.1%) | 93 (93%) | 49 (100%) | 96 (96%) | 98 (99%) | 96 (96%) | 89 (100%) | 78 (73.6%) | 44 (93.6%) |
| rs1393350 | AA | 0 (0%) | 0 (0%) | 0 (0%) | 0 (0%) | 0 (0%) | 0 (0%) | 0 (0%) | 4 (3.8%) | 0 (0%) |
|  | GA | 0 (0%) | 2 (2%) | 0 (0%) | 7 (7%) | 0 (0%) | 5 (5%) | 0 (0%) | 13 (12.3%) | 0 (0%) |
|  | GG | 105 (100%) | 98 (98%) | 49 (100%) | 93 (93%) | 99 (100%) | 95 (95%) | 89 (100%) | 89 (84%) | 47 (100%) |
| rs1126809 | AA | 0 (0%) | 0 (0%) | 0 (0%) | 0 (0%) | 0 (0%) | 0 (0%) | 0 (0%) | 0 (0%) | 0 (0%) |
|  | AG | 0 (0%) | 1 (1%) | 0 (0%) | 7 (7%) | 1 (1%) | 5 (5%) | 0 (0%) | 18 (17%) | 0 (0%) |
|  | GG | 105 (100%) | 99 (99%) | 49 (100%) | 93 (93%) | 98 (99%) | 95 (95%) | 89 (100%) | 88 (83%) | 47 (100%) |

SNP: single nucleotide polymorphism
