# Supplementary material for: Association of pigmentation related-genes polymorphisms and geographic environmental variables in the Chinese population
Source: Hereditas. 2021 Jul 8;158:24. doi: 10.1186/s41065-021-00189-7 (PMC8268332; doi:10.1186/s41065-021-00189-7)
Supplement: Supplementary file 3 — Additional file 3: Supplementary Table 3. The HWE-P value of each SNP in different ethnic groups. [file 41065_2021_189_MOESM3_ESM.docx]

Supplementary Table 3 The HWE-*P* value of each SNP in different ethnic groups

| SNP-ID | Tibetan | Ewenki | Hainan-Han | Hui | Li | Mongolian | Miao | Uighur | Shaanxi-Han |
| --- | --- | --- | --- | --- | --- | --- | --- | --- | --- |
| rs11568737 | 1.000 | 1.000 | 1.000 | 1.000 | 0.137 | 1.000 | 1.000 | 1.000 | 1.000 |
| rs28777 | 0.674 | 0.327 | 1.000 | 0.024 | 0.613 | 0.666 | 1.000 | 0.645 | 1.000 |
| rs183671 | 1.000 | 1.000 | 0.332 | 0.095 | 0.332 | 1.000 | 1.000 | 0.255 | 1.000 |
| rs1042602 | 1.000 | 1.000 | 1.000 | 1.000 | 1.000 | 1.000 | 1.000 | 0.248 | 1.000 |
| rs1393350 | 1.000 | 1.000 | 1.000 | 1.000 | 1.000 | 1.000 | 1.000 | 0.008 | 1.000 |
| rs1126809 | 1.000 | 1.000 | 1.000 | 1.000 | 1.000 | 1.000 | 1.000 | 1.000 | 1.000 |

SNP: single nucleotide polymorphism, HWE: Hardy-Weinberg equilibrium

*P* < 0.05 was considered to be significant.
